# Supplementary material for: The hidden life of Xylella: mining the NCBI Sequence Read Archive reveals potential new species, host plants, and infected areas for this elusive bacterial plant pathogen
Source: Appl Environ Microbiol. 2025 Sep 19;91(10):e00913-25. doi: 10.1128/aem.00913-25 (PMC12542735; doi:10.1128/aem.00913-25)
Supplement: Fig. S1 — Phylogenomic analysis confirming that the newly assembled genomes belong to the genus Xylella. [file aem.00913-25-s0001.pdf]

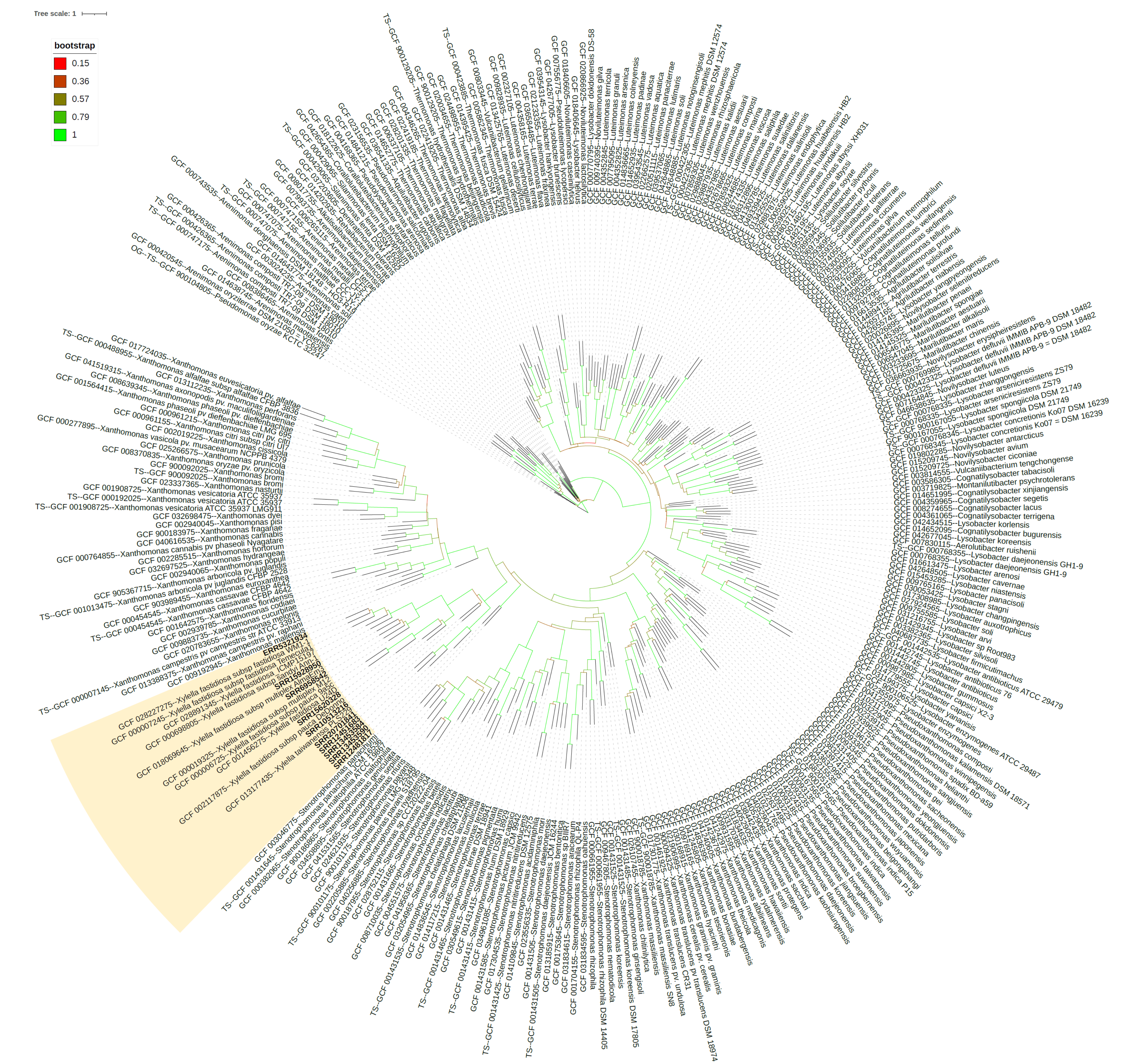

Supplementary Figure S1. Phylogenomic analysis confirming that the newly-assembled genomes belong to the genus *Xylella*. Maximum-likelihood phylogenetic tree based on four conserved protein-coding genes shared between 267 genomes from 28 genera of the *Lysobacteraceae* family, with *Pseudomonas oryzae* as outgroup. Branch support is based on 1000 bootstrap iterations. The new genomes assembled from SRA datasets are indicated in bold and were placed within the genus *Xylella,* highlighted in yellow.
