# Supplementary material for: The hidden life of Xylella: mining the NCBI Sequence Read Archive reveals potential new species, host plants, and infected areas for this elusive bacterial plant pathogen
Source: Appl Environ Microbiol. 2025 Sep 19;91(10):e00913-25. doi: 10.1128/aem.00913-25 (PMC12542735; doi:10.1128/aem.00913-25)
Supplement: Fig. S2 — KEGG pathway completeness in representative Xylella species genomes. [file aem.00913-25-s0002.pdf]

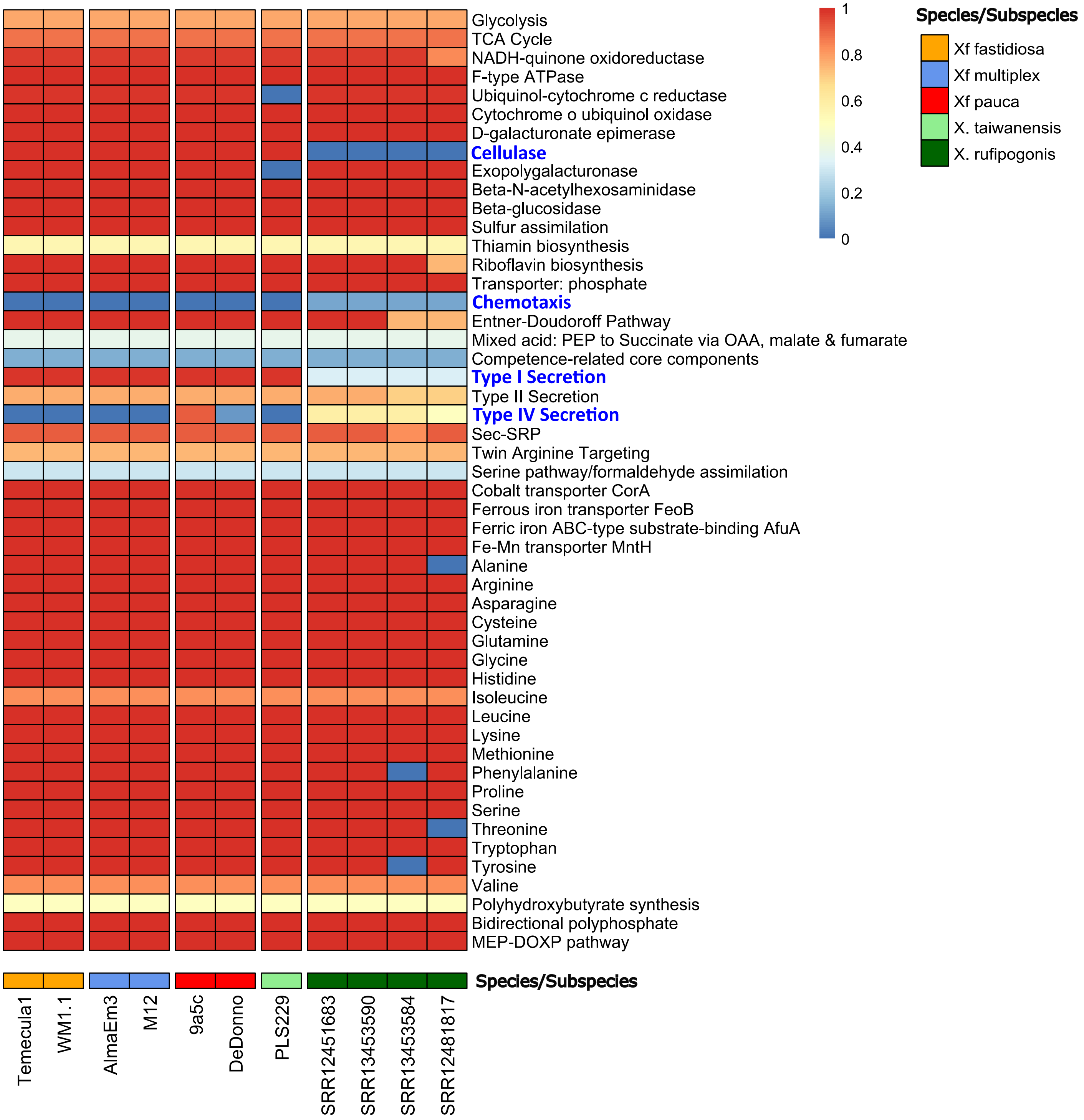

Supplementary Figure S2. KEGG pathway completeness in representative *Xylella* spp. genomes. Heatmap showing the completeness of KEGG pathways/categories determined by KEGG Decoder. Only pathways detected in at least one strain are shown. *Xylella* species and *X. fastidiosa* subspecies are colour-coded as in Fig. 2. KEGG pathways/categories differentially represented in the four *X. rufipogonis* genomes are highlighted in blue.
